# Effectiveness of Tai Chi exercise on balance, falls, and motor function in older adults: a meta-analysis

**DOI:** 10.3389/fmed.2024.1486746

**Published:** 2024-11-05

**Authors:** Liangxing Li, Shoujun Guo, Bing Ding, Jinsong Zhang

**Affiliations:** ^1^Postgraduate School, Harbin Sport University, Harbin, China; ^2^Department of Physical Education and Research, Heilongjiang International University, Harbin, China; ^3^Football Teaching and Research Office, Harbin Sport University, Harbin, China; ^4^Postgraduate School, Harbin Sport University, Harbin, China

**Keywords:** Tai Chi, older adults, falls, balance, physical functional abilities

## Abstract

**Objective:**

This study systematically evaluated Tai Chi’s effects on balance, fall prevention, and physical function in older adults.

**Methods:**

A comprehensive literature search of PubMed, Web of Science, and EMbase databases included randomized controlled trials published up to August 2024. The meta-analysis used RevMan 5.4 and applied the GRADE framework to assess evidence quality.

**Results:**

Twenty-two studies met the criteria. Tai Chi significantly improved balance and physical function, particularly in the Berg Balance Scale (BBS), one-leg standing with eyes closed (OLS-C), and Timed up-and-go test (TUG). Fear of falling (FOF) also reduced, though changes in Tinetti Balance Test (TBT) and Short Physical Performance Battery (SPPB) were not statistically significant.

**Conclusion:**

Tai Chi effectively enhances balance and physical function in older adults, with notable potential for fall risk reduction. While some tests showed no significant effect, overall results support Tai Chi as a valuable intervention to promote older adults’ health.

**Systematic review registration:**

https://inplasy.com/inplasy-2024-8-0082/.

## 1 Introduction

The aging global population presents an impending public health challenge, with projections suggesting that by mid-century, approximately 20% of the world’s population will be aged 60 years or older (1, 2). Age-related declines in physical function and coordination significantly elevate the risk of falls, which, in turn, detrimentally impact quality of life (3, 4). Falls in older adults are associated not only with physical injuries but also with psychological consequences, including increased anxiety, depression, and social isolation (5, 6). Moderate exercise serves as an effective preventive strategy, promoting physical health while simultaneously alleviating loneliness and enhancing social connectedness through group participation (7). Tai Chi, an ancient Chinese martial art recognized for its health-promoting qualities, is particularly well-suited for older adults seeking sustainable exercise options (8). Its low-intensity movements, ease of learning, and group-oriented nature make Tai Chi an accessible and appealing option for this demographic (9–11). Through gentle, flowing movements, Tai Chi supports cardiovascular health, strengthens muscles, and enhances joint flexibility, offering a holistic approach to physical well-being (11–13). Additionally, practicing Tai Chi in a group setting fosters mental well-being by reducing loneliness and enhancing a sense of belonging, which together may contribute to a lowered risk of falls. While extensive evidence supports Tai Chi’s benefits in enhancing physical function and balance among older adults, the results across studies are not entirely consistent (14, 15). Some specific outcome measures have received limited examination, and this may reduce the statistical power in meta-analytic evaluations, thereby obscuring potential significant effects (16, 17). This meta-analysis seeks to rigorously reevaluate the impact of Tai Chi on the health of older adults, with a focus on identifying differential effects across various subgroups to pinpoint critical factors that may influence its effectiveness (18). By implementing a more stringent quantitative approach, this study aims to provide refined, evidence-based recommendations for clinical practice, maximizing Tai Chi’s potential in enhancing not only physical but also mental and cognitive functions in the elderly population (19, 20).

## 2 Materials and methods

This meta-analysis was performed and reported in accordance with PRISMA guidelines and registered with the INPLASY (INPLASY202480082).

### 2.1 Inclusion criteria

#### 2.1.1 Population

Individuals aged 60 years and above, who have not participated in Tai Chi within the past year, regardless of ethnicity or nationality. Exclusions include those with serious acute or chronic conditions such as stroke, transient ischemic attacks, unstable angina, decompensated heart failure, Parkinson’s disease, multiple sclerosis, inner ear infections, Meniere’s disease, cognitive impairments, osteoporosis, rheumatoid arthritis, osteoarthritis, severe psychiatric disorders, or those unable to cooperate.

#### 2.1.2 Intervention

The intervention group will participate in Tai Chi as well as Tai Chi combined with other activities (such as painting or singing) that do not affect the outcomes.

#### 2.1.3 Comparison

The control group will engage in conventional exercises or physical therapy.

#### 2.1.4 Outcome measures

The study outcomes include measures of balance, fall risk, and physical function, assessed using tools such as the BBS, TBT, OLS-C, TUG, FES, FOF, and SPPB, as detailed in Table 1 (6, 21–26).

**Table 1:** Outcome measurement tools and descriptions.

Tool	Description
(A) Balance testing	
BBS	The scale includes 14 items, each scored from 0 to 4, with a total score range of 0–56. Higher scores indicate better balance ability. The test includes a range of activities from sitting in a chair to standing on one leg. The BBS test takes approximately 10–15 min to complete and requires a chair, stopwatch, ruler, and step stool
TUG	The TUG test comprises three stages: standing up, walking 3 m, turning around, and returning. Timing begins when the participant stands up and stops when they return and sit back down. The results are used to assess balance and gait changes in older adults before and after intervention
OLS-C	The one-leg stance test with eyes closed is performed to assess balance by eliminating visual input, requiring participants to rely solely on the vestibular system and proprioceptors. OLS-C is commonly used to evaluate balance ability and postural control
(B) Fall assessment	
FES	FES is used to assess the level of fear of falling during daily activities. Participants rate their fear of falling for specific activities, with a scale from 1 (slightly concerned) to 4 (very concerned). The total score ranges from 16 to 64, with higher scores indicating a greater fear of falling
FOF	Describes the FOF experienced by older adults or patients with conditions like osteoporosis. FOF often leads individuals to reduce physical activity to avoid the risk of falling, which can affect their daily life and health
Fall Efficacy Test	The Fall Efficacy Test is a tool to evaluate an individual’s confidence in avoiding falls during daily activities. It typically includes a series of activities where participants self-rate their fall risk for each activity. Higher scores generally indicate greater fear or concern about falling
TBT	The TBT is used to assess balance and gait in older adults. The test comprises 16 items, with 9 items assessing balance and 7 items assessing gait. The total score is 28, with lower scores indicating a higher risk of falling (scores below 18 represent high risk, 19–23 represent moderate risk, and scores above 24 represent low risk)
(C) Physical function assessment	
SPPB	The SPPB is a standardized tool used to assess overall physical function in older adults. The SPPB includes three components: a balance test, a gait speed test (typically a 4-meter walk), and a strength test (evaluated by timing five consecutive chair stands). Each component is scored from 0 to 4, with a total score range of 0–12. Lower scores indicate more severe physical impairment
Chair Stand Test	The Chair Stand Test assesses lower body strength by measuring how many times a participant can rise from a seated position to standing and back as quickly as possible within a set time. This test is commonly used to evaluate muscle strength and functional activity capacity

#### 2.1.5 Study design

Randomized controlled trials.

### 2.2 Exclusion criteria

#### 2.2.1 Duplicate publications

Studies that are duplicate publications of previous research will be excluded to prevent redundancy.

#### 2.2.2 Inaccessibility of full texts

Studies for which the full texts are not available will be excluded, as full text review is essential for quality assessment.

#### 2.2.3 Lack of usable data

Studies that do not provide sufficient data for extraction or have incomplete datasets that cannot be used for meta-analysis.

#### 2.2.4 Type of publication

Reviews, conference abstracts, and case reports will be excluded, as they often do not provide original research data or detailed methodologies.

#### 2.2.5 Intervention criteria

Studies without a pure Tai Chi intervention group will be excluded. This includes studies where Tai Chi is not the main or sole intervention or is mixed with other therapeutic modalities that could confound results.

### 2.3 Search strategy

To conduct a comprehensive and systematic review of the relevant literature, we will search the PubMed, Web of Science, and EMbase databases, covering the period from the inception of each database until August 2024. The search strategy will employ a combination of controlled vocabulary (e.g., MeSH terms) and free-text keywords, including: Tai-ji, Tai Chi, Tai Chi Chuan, T’ai Chi, Age, Older, Elderly, Senior, Fall, Falling, Falls, Accidental Fall, Physical Functional Ability, Physical Functional Performance, Functional Performance, Random, Randomized Controlled Trial, and RCT. The search terms will be refined iteratively based on the initial results to optimize retrieval accuracy. In addition to electronic database searches, manual searches will be conducted to identify relevant studies, and reference lists of included articles will be scrutinized to ensure comprehensive coverage of the field. The specific search strategy for PubMed is detailed in Table 2.

**Table 2:** PubMed search strategy.

Search strategy
#1 “Tai Ji”[Mesh] #2 “Tai-ji” OR “Tai Chi”OR “Tai Chi Chuan” OR “T’ai Chi” OR “Tai-ji”[All fields] #3 #1 OR #2 #4 “Age”[Mesh] #5 “Older”OR “Elderly” OR “Senior” [All fields] #6 #4 OR #5 #7 “Fall”[Mesh] #8 “Falling” OR “Falls” OR “Accidental Fall” [All fields] #9 #7 OR #8 #10 “Physical functional ability”[Mesh] #11 “Physical Functional Performances” OR “Functional Performances”[All fields] #12 #10 OR #11 #13 “Random”[Mesh] #14 “Randomized controlled trial” OR “RCT”[All fields] #15 #13 OR #14 #16 #3 AND #6 AND #9 AND #12 AND #15

### 2.4 Quality assessment of included studies

The quality of the studies included in this review will be independently evaluated by two researchers, following the guidelines recommended by the Cochrane Handbook version 5.1.0 for assessing risk of bias. The evaluation will address six key criteria:

1. Random Sequence Generation: Assessment of whether an appropriate method was employed to generate the randomization sequence, ensuring the randomness of participant allocation.
2. Allocation Concealment: Determination of whether the allocation process was adequately concealed to prevent any foreknowledge of group assignments prior to allocation, thereby reducing selection bias.
3. Blinding: Evaluation of the implementation of blinding for participants, intervention personnel, and outcome assessors, to minimize performance and detection bias.
4. Completeness of Outcome Data: Examination of the integrity of the outcome data, including the reporting of pre and post-intervention measurements, the rate of loss to follow-up or withdrawals (with an attrition rate below 10% generally considered acceptable), and whether an intention-to-treat (ITT) analysis was conducted for missing data.
5. Selective Outcome Reporting: Review of whether all pre-specified outcomes were reported, with particular attention to safety-related outcomes (e.g., adverse events such as falls resulting in death or disability) and any negative results.
6. Other Potential Sources of Bias: Consideration of additional sources of bias, such as premature trial termination or baseline imbalances that could affect the validity of the study results.

The risk of bias in the RCTs was rigorously evaluated using the revised Cochrane Risk of Bias tool (RoB2), which examines several critical domains: the randomization process, deviations from intended interventions, missing outcome data, outcome measurement, and the selection of reported results. To ensure accuracy and objectivity, discrepancies in assessment were addressed through discussions between two authors to reach a consensus; if consensus was not achieved, a third author was consulted. Each domain was classified into one of three categories: “low,” “some concerns,” or “high” risk of bias. The overall risk of bias for each trial was determined by the highest level of risk observed in any domain. Additionally, the quality of evidence supporting our outcomes was systematically appraised using the Grading of Recommendations Assessment, Development, and Evaluation (GRADE) framework, ensuring a robust evaluation of the evidence base. This comprehensive assessment process aligns with the stringent standards required for evidence-based practice, particularly in the fields of sports and elderly rehabilitation, where precise and reliable data interpretation is critical for clinical decision-making.

### 2.5 Data extraction

Data extraction will be independently performed by two researchers, who will review the literature according to the pre-defined inclusion and exclusion criteria. A standardized data extraction form will be utilized to systematically collect the following information:

1. Basic Information: Including the first author, year of publication, and the mean age and standard deviation of the participants (M ± SD).
2. Sample Size: The total number of participants in each study.
3. Study Design and Interventions: Comprehensive documentation of the study design, detailed descriptions of the interventions, including duration and frequency, and the specific outcome measures used.

Any discrepancies identified during the data extraction process will be resolved through discussion between the researchers until consensus is achieved. For studies with incomplete reporting, the researchers will attempt to contact the original authors to request the missing data. If the required information cannot be obtained, the study will be excluded from the analysis.

### 2.6 Statistical analysis

Meta-analysis will be performed using RevMan 5.4 software. The primary effect parameter will be the difference between post-intervention values and baseline measurements (referred to as the difference score). This will be calculated using the following formula:


$$S^{2}=S_{1}^{2}+S_{2}^{2}-2*R*S_{1}*S_{2;}$$

$$M=|M_{1}-M_{2}|$$

where *M* represents the effect mean, *M*_1_ is the baseline mean, *M*_2_ is the post-intervention mean, *S* is the effect standard deviation, *S*_1_ is the baseline standard deviation, *S*_2_ is the post-intervention standard deviation, and R is a constant (either 0.4 or 0.5) used for the calculation.

For categorical data, the risk ratio (RR) will be used as the effect size, with each effect size reported as an RR with a 95% confidence interval (CI).

For continuous variables, the weighted mean difference (WMD) will be used as the summary statistic if all studies measure the same outcome using identical scales. If different scales are used across studies to assess the same outcome, the standardized mean difference (SMD) will be employed. All analyses will include a 95% confidence interval (95% CI).

Statistical heterogeneity will be assessed using the chi-square test and quantified with the *I*^2^ statistic and *p* value. If *p* > 0.1 and *I*^2^ < 50%, a fixed-effect model will be applied. If *p* < 0.1 or *I*^2^ ≥ 50%, and the studies are considered clinically homogeneous, a random-effects model will be used. If heterogeneity is substantial, a descriptive analysis will be conducted.

### 2.7 Quality of evidence assessment using the GRADE framework

The quality of evidence was systematically evaluated using the GRADE (Grading of Recommendations, Assessment, Development, and Evaluation) framework, a widely recognized tool in evidence-based practice (27). The GRADE approach examines five critical domains: risk of bias, publication bias, indirectness, imprecision, and inconsistency. Each domain is rigorously analyzed to determine its impact on the overall confidence in the evidence. Following this comprehensive assessment, each outcome is categorized into one of four levels: high, moderate, low, or very low quality. This structured evaluation ensures a robust and transparent appraisal of the evidence, which is crucial for making informed clinical decisions, particularly in the fields of sports and elderly rehabilitation, where the precision of evidence directly influences patient care and therapeutic outcomes.

## 3 Results

### 3.1 Literature search outcomes and characteristics of included studies

**Figure 1:**
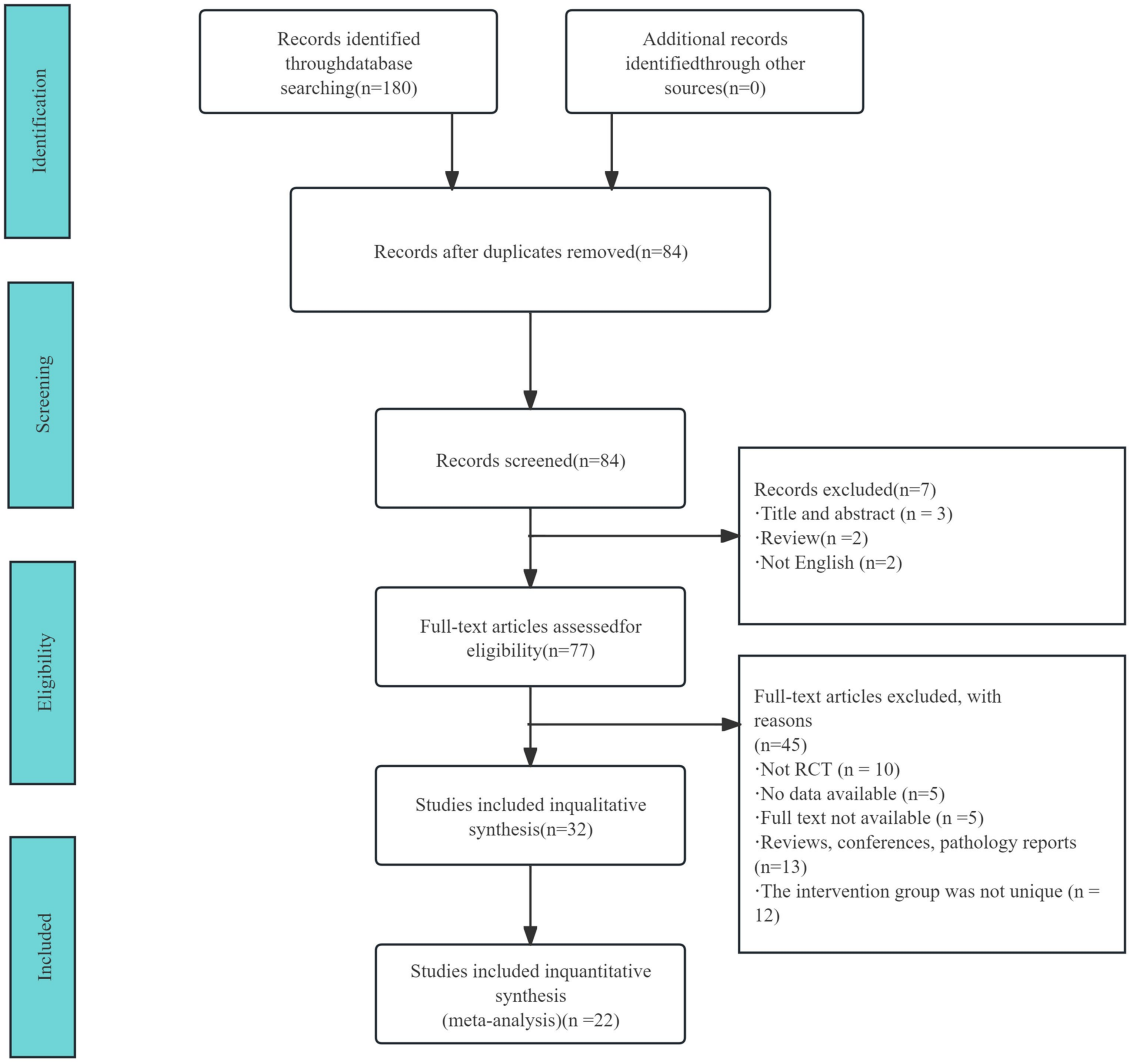
Literature screening process and results.

The literature screening process and its outcomes are illustrated in Figure 1. An initial search identified 180 studies. After removing duplicates, 84 studies remained. Of these, 45 studies were excluded for the following reasons: they were non-randomized controlled trials, duplicate publications, lacked access to full-text versions, or did not provide usable data. Consequently, 22 studies, encompassing a total of 2,170 participants, were included in the final analysis. The basic characteristics of these included studies are summarized in Table 3. Details of the excluded references are provided in Supplementary material.

**Table 3:** Basic characteristics of included studies.

First author and year	Age/years	Sample size		Intervention measures	Exercise details			Outcome measures
		Intervention group	Control group		Duration per session	Frequency per week	Duration	
Li 2004	75.30 ± 7.8	62	56	Tai Chi	60 min	3 times	24 weeks	⑨
Choi 2005	77.8 ± 6.9	29	30	Tai Chi	20 min	3 times	12 weeks	③⑥
Li 2005	77.48 ± 4.95	125	131	Tai Chi	60 min	3 times	24 weeks	①③④⑤
Li 2008	65.2 ± 2.9	25	25	Tai Chi	60 min	4 times	16 weeks	③
Pereira 2008	68 ± 5	38	39	Tai Chi	50 min	3 times	12 weeks	③
Logghe 2009	77.5 ± 4.7	138	131	Tai Chi	60 min	2 times	13 weeks	①⑤
Huang 2010	71.5 ± 1.0	31	47	Tai Chi	40 min	3 times	20 weeks	⑦
Kruse 2012	68.9 ± 5.1	48	48	Tai Chi	60 min	2 times	24 weeks	⑤
Manor 2014	87 ± 7	29	28	Tai Chi	60 min	2 times	12 weeks	①④⑧⑨
Saravanakumar 2014	83.8 ± 8.0	11	11	Tai Chi	30 min	2 times	14 weeks	①
Hwang 2016	72.0 ± 8.1	228	228	Tai Chi	60 min	7 times	24 weeks	②⑤
Qian 2017	64.77 ± 4.00	96	96	Tai Chi	50 min	5 times	24 weeks	③④
Hosseini 2018	69.1 ± 5.3	30	30	Tai Chi	55 min	2 times	8 weeks	②④⑤
Mortazavi 2018	67.63 ± 5.32	27	26	Tai Chi	30 min	3 times	10 weeks	⑦
You 2018	75 ± 8	28	26	Tai Chi	60 min	2 times	12 weeks	⑥⑧
Penn 2019	75.1 ± 7.6	20	15	Tai Chi	30 min	3 times	8 weeks	①④
Sun 2019	64.12 ± 3.21	12	13	Tai Chi	60 min	5 times	16 weeks	③
Zhang 2019	70.3 ± 5.1	16	16	Tai Chi	60 min	3 times	12 weeks	③④⑤
Chen 2021	76.4 ± 5.9	36	32	Tai Chi	60 min	2 times	12 weeks	③
Okuyan 2021	74.21 ± 6.93	20	22	Tai Chi	35–40 min	2 times	12 weeks	②
Tang 2021	68.86 ± 10.26	25	24	Tai Chi	60 min	2 times	12 weeks	①③④⑤
Zong 2022	68.58 ± 4.55	11	11	Tai Chi	60 min	4 times (first 8 weeks), 3 times (weeks 9–24)	24 weeks	①

① Berg Balance Scale (BBS); ② Tinetti Balance Test (TBT); ③ One-Legged Stance with Eyes Closed (OLS-C); ④ Timed Up and Go (TUG); ⑤ Falls Efficacy Scale (FES); ⑥ Falls Efficacy; ⑦ Fear of Falling (FOF); ⑧ Short Physical Performance Battery (SPPB); ⑨ Chair Stand Time.

### 3.2 Methodological quality assessment of included studies

**Figure 2:**
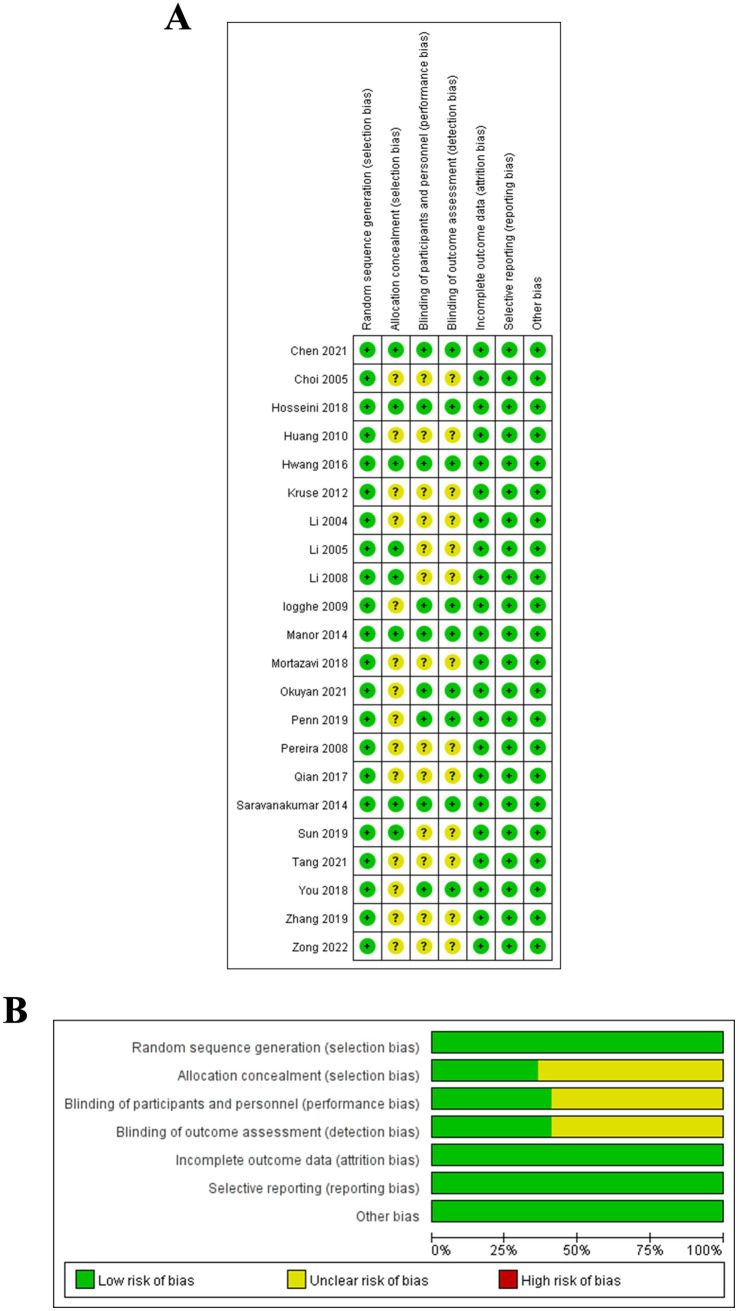
**(A)** Methodological quality distribution of the included 22 RCTs; **(B)** Bias risk graph and summary for the included 22 RCTs.

All 22 included RCTs reported baseline characteristics of participants and explicitly stated the use of randomization (28–49). Seven RCTs provided details on allocation concealment, and 11 RCTs reported the implementation of blinding. Data reporting was complete across all 22 RCTs, with adequate descriptions of any missing data or the reasons for their absence. Furthermore, all 22 RCTs demonstrated selective outcome reporting, and no additional sources of bias were identified in these studies. The detailed results of the quality assessment are illustrated in Figure 2.

### 3.3 Meta-analysis results

#### 3.3.1 BBS test

**Figure 3:**
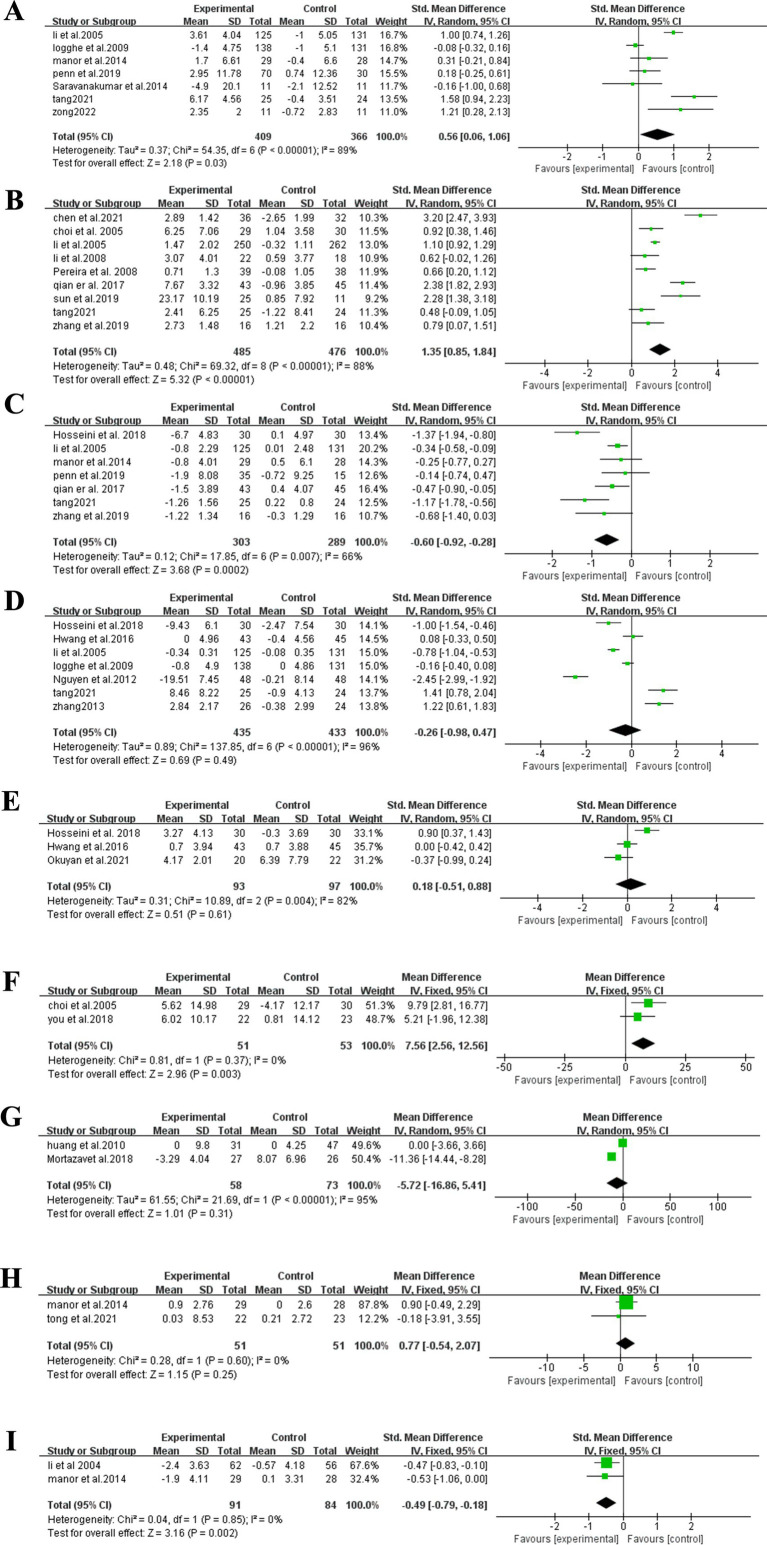
Meta-analysis outcomes. **(A)** BBS; **(B)** OLS-C; **(C)** TUG; **(D)** FES; **(E)** TBT; **(F)** Fall efficacy; **(G)** FOF; **(H)** SPPB; **(I)** Chair stand test.

Seven studies, encompassing a total of 775 participants, compared the BBS scores between the Tai Chi and control groups (27). Given the heterogeneity among the included studies, a random-effects model was applied. The analysis revealed that the Tai Chi group demonstrated significantly higher BBS scores compared to the control group, with a statistically significant difference (SMD = 0.56, 95% CI: 0.06 to 1.06, *p* = 0.03) (Figure 3A).

#### 3.3.2 OLS-C test

Nine studies, encompassing a total of 961 participants, compared the OLS-C scores between the Tai Chi and control groups (29, 34, 35, 42, 45–49). Given the heterogeneity among the included studies, a random-effects model was applied. The analysis revealed that the Tai Chi group demonstrated significantly higher OLS-C scores compared to the control group, with a statistically significant difference (SMD = 1.35, 95% CI: 0.85–1.84, *p* < 0.00001) (Figure 3B).

#### 3.3.3 TUG test

Seven studies, encompassing a total of 592 participants, were analyzed to compare TUG test performance between the Tai Chi and control groups (29, 30, 32, 34, 39, 47, 49). Given the heterogeneity among the included studies, a random-effects model was applied. The analysis revealed that the Tai Chi group demonstrated significantly higher TUG scores compared to the control group, with a statistically significant difference (SMD = −0.60, 95% CI: −0.92 to −0.28, *p* = 0.0002) (Figure 3C).

#### 3.3.4 FES test

Seven studies, encompassing a total of 868 participants, were analyzed to compare FES test performance between the Tai Chi and control groups (29, 33, 34, 39, 40, 43, 49). Given the heterogeneity among the included studies, a random-effects model was applied. The results indicated no statistically significant difference between the Tai Chi and control groups in the FES test (SMD = −0.29, 95% CI: −0.98 to 0.42, *p* = 0.49) (Figure 3D).

#### 3.3.5 TBT test

Three studies, encompassing a total of 190 participants, were analyzed to compare TBT performance between the Tai Chi and control groups (39, 41, 43). Given the heterogeneity among the included studies, a fixed-effects model was applied. The results indicated no statistically significant difference between the Tai Chi and control groups in the TBT test (MD = 0.65, 95%CI: −2.40 to 3.70, *p* = 0.53) (Figure 3E).

#### 3.3.6 Fall efficacy test

Two studies, encompassing a total of 104 participants, were analyzed to compare fall efficacy test results between the Tai Chi and control groups (35, 36). Given the heterogeneity among the included studies, a fixed-effects model was applied. The analysis revealed that the Tai Chi group demonstrated significantly higher fall efficacy test compared to the control group, with a statistically significant difference (MD = 7.56, 95%CI: 2.56–12.56, *p* = 0.003) (Figure 3F).

#### 3.3.7 FOF test

Two studies, encompassing a total of 131 participants, were analyzed to compare FOF test results between the Tai Chi and control groups (37, 38). Given the heterogeneity among the included studies, a fixed-effects model was applied. The results indicated no statistically significant difference between the Tai Chi and control groups in the FOF test (MD = −5.72, 95% CI: −16.86 to 5.41, *p* = 0.31) (Figure 3G).

#### 3.3.8 SPPB test

Two studies, encompassing a total of 102 participants, were analyzed to compare SPPB test results between the Tai Chi and control groups (32, 36). Given the heterogeneity among the included studies, a fixed-effects model was applied. The results indicated no statistically significant difference between the Tai Chi and control groups in the SPPB test (MD = 0.77, 95% CI: −0.54 to 2.07, *p* = 0.25) (Figure 3H).

#### 3.3.9 Chair stand test

Two studies, encompassing a total of 175 participants, were analyzed to compare chair stand test results between the Tai Chi and control groups (32, 44). Given the heterogeneity among the included studies, a random-effects model was applied. The analysis revealed that the Tai Chi group demonstrated significantly higher Chair Stand Test compared to the control group, with a statistically significant difference (SMD = −0.49, 95% CI: −0.79 to −0.19, *p* = 0.002) (Figure 3I).

### 3.4 GRADE assessment of outcome evidence

The quality of evidence for the outcomes analyzed in this study was evaluated using the GRADE (Grading of Recommendations, Assessment, Development, and Evaluation) framework. The assessment indicated that the evidence quality for each outcome was rated as low. Several factors likely contributed to this rating, including the risk of bias inherent in the study designs, inconsistency in results across studies, imprecision due to small sample sizes, and concerns related to indirectness or publication bias. Additionally, the observed variability in the incidence and progression of falls, balance issues, and physical functioning among the elderly population further exacerbates these inconsistencies. For a detailed summary of the evidence quality assessed in this meta-analysis, please refer to Table 4 (27).

**Table 4:** The GRADE tool for the pooled results in the patients after concurrent training.

Outcome	Illustrative comparative risks (95% CI)*	Number of participants (studies)	Certainty of the evidence
	Corresponding risk		
	With exercise		
BBS	The mean BBS score post-intervention was 0.56 points higher (0.06 lower to 1.06 higher) compared to usual exercise	775 (7 studies)	Low*†‡+
OLS-C	The mean OLS-C test score post-intervention was 1.35 points higher (0.85 lower to 1.84 higher) compared to usual exercise	961 (9 studies)	Low*†‡+
TUG	The mean TUG test score post-intervention was 0.60 points lower (−0.92 lower to −0.28 higher) compared to usual exercise	592 (7 studies)	Low*†‡+
FES	The mean FES score post-intervention was 0.29 points lower (−0.98 lower to 0.42 higher) compared to usual exercise	868 (7 studies)	Low*†‡+
TBT	The mean TBT score post-intervention was 0.65 points higher (−2.40 lower to 3.70 higher) compared to usual exercise	190 (3 studies)	Low*†‡+
Fall Efficacy	The mean fall efficacy score post-intervention was 7.56 points higher (2.56 lower to 12.56 higher) compared to usual exercise	104 (2 studies)	Low*†‡+
FOF	The mean FOF score post-intervention was 5.72 points lower (−16.86 lower to 5.41 higher) compared to usual exercise	131 (2 studies)	Low*†‡+
SPPB	The mean SPPB score post-intervention was 0.77 points higher (−0.54 lower to 2.07 higher) compared to usual exercise	102 (2 studies)	Low*†‡+
Chair Stand	The mean chair stand time post-intervention was 0.49 s shorter (−0.79 lower to −0.19 higher) compared to usual exercise	175 (2 studies)	Low*†‡+

High quality: Further research is very unlikely to change our confidence in the estimate of effect.

Moderate quality: Further research is likely to have an important impact on our confidence in the estimate of effect and may change the estimate.

Low quality: Further research is very likely to have a significant impact on our confidence in the estimate of effect and is likely to change the estimate.

Very low quality: We have a high degree of uncertainty about the estimate.

^*^Design limitations.

^†Publication bias.^

^‡Imprecision.^

^+^Heterogeneity.

The assumed risk is based on the median control group risk across studies, as detailed in the footnotes. The corresponding risk (and its 95% confidence interval) is calculated based on the assumed risk in the comparison group and the relative effect of the intervention (and its 95% CI). Control group risk estimates are derived from pooled estimates of control groups, with the relative effect determined from available case analysis.

## 4 Discussion

### 4.1 Study findings

This meta-analysis systematically assessed the effects of Tai Chi on improving balance, reducing fear of falling, and enhancing physical function in older adults. The findings indicate that Tai Chi significantly improved scores on the BBS and extended the duration of OLS-Close, demonstrating its positive impact on balance enhancement in this population. Furthermore, Tai Chi was associated with significant improvements in the TUG test and Fall Efficacy test, underscoring its potential to enhance physical function and reduce fall risk among older adults. However, the results for the TBT, FES, FOF, and SPPB did not achieve statistical significance, suggesting that Tai Chi’s effects may vary depending on the specific balance and functional measures used.

#### 4.1.1 The impact of Tai Chi on balance in older adults

This study assessed the effects of Tai Chi on balance using the BBS, TUG, and OLS-C tests. Our findings in the TUG test are consistent with those of Li et al. (50), who also reported that Tai Chi significantly improves dynamic balance in older adults. This further supports the role of Tai Chi in reducing fall risk among the elderly (50).

In the OLS-C test, our study found that the Tai Chi group outperformed the control group, indicating that Tai Chi significantly enhances the ability to stand on OLS-C in older adults. Song et al. (51) noted that while Tai Chi has a significant impact on dynamic balance, its effects on certain static balance tests may be more limited, which contrasts with our findings. This discrepancy might be due to the inclusion of a more heterogeneous population with varying health conditions in Song et al.’s study, leading to greater variability in results, whereas our study sample was more homogeneous, potentially reducing such variability (51).

Regarding the BBS test, our results demonstrated that Tai Chi significantly improved balance in older adults, aligning with the findings of Wang et al. (52). Wang’s study highlighted that Tai Chi, particularly the 24-form simplified Yang style, has moderate to significant effects on improving both static and overall balance in older adults (52). This supports our conclusion that Tai Chi effectively enhances balance in this population. However, compared to Song et al. (51), our study observed more pronounced improvements on the BBS. Song et al. suggested that the balance-enhancing effects of Tai Chi might vary across different tests, with weaker effects noted for dynamic balance. These differences could be attributed to variations in the form of Tai Chi practiced, training frequency, or differences in participants’ health status and baseline balance abilities across the studies.

#### 4.1.2 The impact of Tai Chi on falls in older adults

Our study found that Tai Chi significantly reduced fear of falling in older adults, as measured by the FES, aligning with the findings of Zhang et al. (53). Zhang and colleagues demonstrated through meta-analysis that Tai Chi practice significantly improves fall efficacy in older adults, with particularly strong effects in the short term (53). Although we observed some improvement in the Tai Chi group compared to the control group in the TBT, this difference did not reach statistical significance. This suggests that while Tai Chi may enhance balance to some extent, its effect under the conditions of our study was not statistically significant. Our findings show some variation compared to other studies. For example, Wang et al. (52) conducted a meta-analysis indicating that Tai Chi has a moderate effect on improving balance in older adults, particularly in static balance tests. Wang’s study highlighted the importance of high-frequency training, whereas the training frequency in our study was lower, which may explain the difference in effects observed (52).

Additionally, we explored the impact of Tai Chi on FOF. Our results showed that the Tai Chi group performed significantly better on the FOF test compared to the control group, consistent with findings from Del-Pino-Casado et al. (54). Their systematic review and meta-analysis found that Tai Chi effectively reduces fall risk and improves related mental health outcomes in older adults (54). However, the extent of improvement in our study differed slightly from that reported by Logghe et al. (55). Logghe’s research suggested that while Tai Chi positively impacts fear of falling in older adults, its long-term effects are less pronounced compared to other interventions (55). These discrepancies may be due to differences in study design, sample characteristics, and intervention duration.

#### 4.1.3 The impact of Tai Chi on physical functional abilities in older adults

Our study found that Tai Chi had a limited overall effect on improving physical function in older adults as measured by the SPPB test, a finding consistent with Wu et al. (56). Wu’s research suggested that while Tai Chi significantly enhances balance and reduces fall rates, its impact on SPPB scores is minimal, possibly due to the SPPB’s limited assessment of dynamic balance (56).

Additionally, we evaluated the impact of Tai Chi on the chair stand test results in older adults. The findings showed a significant improvement in the Tai Chi group, indicating that Tai Chi positively influences physical functional abilities in this population. These results are in line with those of Park et al. (57), whose systematic review and meta-analysis demonstrated that Tai Chi can significantly enhance physical functional abilities in older adults, including improvements in chair stand test performance. This further supports the effectiveness of Tai Chi in enhancing lower limb strength and functional fitness in this population (57).

### 4.2 Potential physiological mechanisms of Tai Chi on balance and physical function in the elderly

Tai Chi supports postural control by reinforcing the visual, proprioceptive, and vestibular systems, all of which are vital for maintaining balance. As individuals age, reduced visual acuity and environmental sensitivity impair balance (58). Tai Chi enhances visual feedback through controlled gaze adjustments, which helps stabilize posture (59). The proprioceptive system, which monitors muscle and joint positions, is strengthened by Tai Chi’s slow, continuous motions, improving coordination and dynamic balance (60). This is particularly beneficial for older adults, allowing better responses to sudden balance disruptions. The vestibular system, responsible for sensing head position and acceleration, is also activated through Tai Chi’s rotational movements and footwork, boosting adaptability and response to balance challenges. Furthermore, Tai Chi exercises enhance lower limb strength by engaging the quadriceps and stabilizing knee joints, which helps reduce gait instability (24). These improvements support older adults in maintaining postural balance during daily activities, thereby lowering fall risks significantly.

### 4.3 Feasibility analysis of implementing Tai Chi in the healthcare system

Assessing the feasibility of incorporating Tai Chi into healthcare requires considering various factors. First, qualified Tai Chi instructors need a solid background in exercise physiology and geriatrics, as well as specialized teaching skills, which could strain finances for resource-limited institutions. However, as Tai Chi requires minimal equipment, institutions could train existing staff or collaborate with local practitioners to reduce costs and facilitate integration. Second, Tai Chi’s low-impact, gentle nature is ideal for elderly patients who prioritize safety and comfort, making it feasible as a long-term intervention. Its adaptable frequency and duration enhance patient adherence and reduce the burdens of intense exercise. Finally, while Tai Chi does not require large equipment, it does need spacious, level areas for safe practice. For facilities with limited indoor space, using outdoor or public areas with appropriate safety measures can meet these spatial needs without extra costs.

### 4.4 The potential of Tai Chi for holistic health promotion in elderly care

Tai Chi, an exercise regimen characterized by low-intensity movements and restorative attention practices, has demonstrated significant benefits in enhancing cognitive function and physical flexibility among older adults. Additionally, it fosters improved mental health and social connectedness. The slow, deliberate motions integral to Tai Chi practice promote attentional restoration, making this exercise particularly beneficial for individuals frequently engaged in multitasking or subjected to high-stress environments. This practice is supported by Attention Restoration Theory (ART), which suggests that alternating between rest and focused activities helps replenish cognitive resources and reduce psychological fatigue (61). Tai Chi’s benefits extend well beyond physical health, encompassing improvements in mental and emotional well-being. Engagement in group-based Tai Chi activities provides older adults with beneficial social interactions, which have been shown to foster emotional resilience and psychological flexibility (62). In contrast to traditional treatment-centered approaches, Tai Chi emphasizes a preventative and health-promoting perspective within elderly care. This integrated approach, which simultaneously addresses physical and mental health needs through communal exercises, underscores Tai Chi’s essential role in contemporary elderly care. It provides a holistic support system that is both preventive and rehabilitative, thus offering comprehensive health benefits to the aging population (61).

### 4.5 Strengths and limitations

A key strength of our research lies in the integration of recent RCTs into our analysis, supported by a comprehensive sensitivity assessment. Nevertheless, our study presents several limitations. First, the reliance on predominantly English-language publications selected according to specific criteria may have introduced selection bias. Second, our findings are confined to standard Tai Chi programs and do not encompass personalized program variations. This limitation underscores the necessity for further research into the efficacy of individualized Tai Chi programs in the context of elderly rehabilitation. Additionally, our study population was restricted to healthy older adults, thereby excluding those with significant comorbidities. While sensitivity and subgroup analyses were conducted, potential heterogeneity might still influence the robustness of our findings. To broaden the applicability of future research, it would be advantageous to include elderly individuals with diverse health conditions. Finally, although our study compared Tai Chi to traditional, non-Tai Chi exercise modalities, various emerging interventions and technologies may also have implications for improving the quality of life among older adults. Future studies should consider examining the distinctive advantages and limitations of Tai Chi through comparative analyses with other innovative interventions. Such studies could provide a more nuanced understanding of Tai Chi’s role within the broader spectrum of rehabilitation strategies for the elderly.

## 5 Conclusion

Tai Chi has shown substantial preventive effects against falls in older adults, while also enhancing their balance and physical functional abilities. However, the current body of research on physical functional abilities is relatively limited, highlighting the need for further studies in this area. Additionally, more frequent follow-up observations are necessary to better assess the long-term effectiveness of Tai Chi interventions. Given the limitations in both the quantity and quality of the included studies, these findings should be interpreted with caution and warrant further validation through high-quality research.

## Data Availability

The original contributions presented in the study are included in the article/Supplementary material, further inquiries can be directed to the corresponding author.
