# Supplementary material for: Effectiveness of Tai Chi exercise on balance, falls, and motor function in older adults: a meta-analysis
Source: Front Med (Lausanne). 2024 Nov 5;11:1486746. doi: 10.3389/fmed.2024.1486746 (PMC11573509; doi:10.3389/fmed.2024.1486746)
Supplement: Supplementary file 2 [file Table_2.DOCX]

Appendix A

| Serial Number | References |
| --- | --- |
| 1 | DAY L, HILL K D, JOLLEY D, et al. Impact of tai chi on impairment, functional limitation, and disability among preclinically disabled older people: a randomized controlled trial [J]. Arch Phys Med Rehabil, 2012, 93(8): 1400-7. |
| 2 | DAY L, HILL K D, STATHAKIS V Z, et al. Impact of tai-chi on falls among preclinically disabled older people. A randomized controlled trial [J]. J Am Med Dir Assoc, 2015, 16(5): 420-6. |
| 3 | DECHAMPS A, DIOLEZ P, THIAUDIèRE E, et al. Effects of exercise programs to prevent decline in health-related quality of life in highly deconditioned institutionalized elderly persons: a randomized controlled trial [J]. Arch Intern Med, 2010, 170(2): 162-9. |
| 4 | FABER M J, BOSSCHER R J, CHIN A P M J, VAN WIERINGEN P C. Effects of exercise programs on falls and mobility in frail and pre-frail older adults: A multicenter randomized controlled trial [J]. Arch Phys Med Rehabil, 2006, 87(7): 885-96. |
| 5 | FRANSEN M, NAIRN L, WINSTANLEY J, et al. Physical activity for osteoarthritis management: a randomized controlled clinical trial evaluating hydrotherapy or Tai Chi classes [J]. Arthritis Rheum, 2007, 57(3): 407-14. |
| 6 | GALLANT M P, TARTAGLIA M, HARDMAN S, BURKE K. Using Tai Chi to Reduce Fall Risk Factors Among Older Adults: An Evaluation of a Community-Based Implementation [J]. J Appl Gerontol, 2019, 38(7): 983-98. |
| 7 | GATTS S K, WOOLLACOTT M H. Neural mechanisms underlying balance improvement with short term Tai Chi training [J]. Aging Clin Exp Res, 2006, 18(1): 7-19. |
| 8 | GOW B J, HAUSDORFF J M, MANOR B, et al. Can Tai Chi training impact fractal stride time dynamics, an index of gait health, in older adults? Cross-sectional and randomized trial studies [J]. PLoS One, 2017, 12(10): e0186212. |
| 9 | GREENSPAN A I, WOLF S L, KELLEY M E, O'GRADY M. Tai chi and perceived health status in older adults who are transitionally frail: a randomized controlled trial [J]. Phys Ther, 2007, 87(5): 525-35. |
| 10 | HALL C D, MISZKO T, WOLF S L. Effects of Tai Chi intervention on dual-task ability in older adults: a pilot study [J]. Arch Phys Med Rehabil, 2009, 90(3): 525-9. |
| 11 | HARTMAN C A, MANOS T M, WINTER C, et al. Effects of T'ai Chi training on function and quality of life indicators in older adults with osteoarthritis [J]. J Am Geriatr Soc, 2000, 48(12): 1553-9. |
| 12 | HASS C J, GREGOR R J, WADDELL D E, et al. The influence of Tai Chi training on the center of pressure trajectory during gait initiation in older adults [J]. Arch Phys Med Rehabil, 2004, 85(10): 1593-8. |
| 13 | HE J, CHAN S H, LIN J, TSANG H W. Integration of tai chi and repetitive transcranial magnetic stimulation for sleep disturbances in older adults: A pilot randomized controlled trial [J]. Sleep Med, 2024, 122: 35-44. |
| 14 | HE S, MENG D, WEI M, et al. Proposal and validation of a new approach in tele-rehabilitation with 3D human posture estimation: a randomized controlled trial in older individuals with sarcopenia [J]. BMC Geriatr, 2024, 24(1): 586. |
| 15 | HSU C Y, MOYLE W, COOKE M, JONES C. Seated Tai Chi versus usual activities in older people using wheelchairs: A randomized controlled trial [J]. Complement Ther Med, 2016, 24: 1-6. |
| 16 | HSU C Y, MOYLE W, COOKE M, JONES C. Seated T'ai Chi in Older Taiwanese People Using Wheelchairs: A Randomized Controlled Trial Investigating Mood States and Self-Efficacy [J]. J Altern Complement Med, 2016, 22(12): 990-6. |
| 17 | HU X, LYU S, MAO M, et al. Effects of Eight Methods and Five Steps of Tai Chi Practice on Balance Control Among Older Adults [J]. Motor Control, 2021, 25(4): 616-30. |
| 18 | HUANG H C, LIU C Y, HUANG Y T, KERNOHAN W G. Community-based interventions to reduce falls among older adults in Taiwan - long time follow-up randomised controlled study [J]. J Clin Nurs, 2010, 19(7-8): 959-68. |
| 19 | HUANG N, LI W, RONG X, et al. Effects of a Modified Tai Chi Program on Older People with Mild Dementia: A Randomized Controlled Trial [J]. J Alzheimers Dis, 2019, 72(3): 947-56. |
| 20 | HUANG T T, YANG L H, LIU C Y. Reducing the fear of falling among community-dwelling elderly adults through cognitive-behavioural strategies and intense Tai Chi exercise: a randomized controlled trial [J]. J Adv Nurs, 2011, 67(5): 961-71. |
| 21 | HWANG H F, CHEN C Y, WEI L, et al. Effects of Computerized Cognitive Training and Tai Chi on Cognitive Performance in Older Adults With Traumatic Brain Injury [J]. J Head Trauma Rehabil, 2020, 35(3): 187-97. |
| 22 | HWANG H F, CHEN S J, LEE-HSIEH J, et al. Effects of Home-Based Tai Chi and Lower Extremity Training and Self-Practice on Falls and Functional Outcomes in Older Fallers from the Emergency Department-A Randomized Controlled Trial [J]. J Am Geriatr Soc, 2016, 64(3): 518-25. |
| 23 | HWANG H F, TSENG K C, CHEN S J, et al. Effects of home-based computerized cognitive training and tai chi exercise on cognitive functions in older adults with mild cognitive impairment [J]. Aging Ment Health, 2023, 27(11): 2170-8. |
| 24 | IBAñEZ G E, AHMED S, HU N, et al. Acceptability and Feasibility of a Tai Chi/Qigong Intervention for Older People Living With HIV [J]. AIDS Behav, 2024, 28(4): 1291-300. |
| 25 | IRWIN M R, OLMSTEAD R. Mitigating cellular inflammation in older adults: a randomized controlled trial of Tai Chi Chih [J]. Am J Geriatr Psychiatry, 2012, 20(9): 764-72. |
| 26 | IRWIN M R, OLMSTEAD R, BREEN E C, et al. Cognitive behavioral therapy and tai chi reverse cellular and genomic markers of inflammation in late-life insomnia: a randomized controlled trial [J]. Biol Psychiatry, 2015, 78(10): 721-9. |
| 27 | IRWIN M R, OLMSTEAD R, CARRILLO C, et al. Cognitive behavioral therapy vs. Tai Chi for late life insomnia and inflammatory risk: a randomized controlled comparative efficacy trial [J]. Sleep, 2014, 37(9): 1543-52. |
| 28 | IRWIN M R, OLMSTEAD R, MOTIVALA S J. Improving sleep quality in older adults with moderate sleep complaints: A randomized controlled trial of Tai Chi Chih [J]. Sleep, 2008, 31(7): 1001-8. |
| 29 | IRWIN M R, OLMSTEAD R, OXMAN M N. Augmenting immune responses to varicella zoster virus in older adults: a randomized, controlled trial of Tai Chi [J]. J Am Geriatr Soc, 2007, 55(4): 511-7. |
| 30 | IRWIN M R, PIKE J L, COLE J C, OXMAN M N. Effects of a behavioral intervention, Tai Chi Chih, on varicella-zoster virus specific immunity and health functioning in older adults [J]. Psychosom Med, 2003, 65(5): 824-30. |
| 31 | JIA X, JIANG C, TAO J, et al. Effects of core strength training combined with Tai Chi Chuan for the musculoskeletal system and cardiopulmonary function in older adults: A study protocol for a randomized controlled trial [J]. Medicine (Baltimore), 2018, 97(35): e12024. |
| 32 | JIAYUAN Z, XIANG-ZI J, LI-NA M, et al. Effects of Mindfulness-Based Tai Chi Chuan on Physical Performance and Cognitive Function among Cognitive Frailty Older Adults: A Six-Month Follow-Up of a Randomized Controlled Trial [J]. J Prev Alzheimers Dis, 2022, 9(1): 104-12. |
| 33 | JUDGE J O, LINDSEY C, UNDERWOOD M, WINSEMIUS D. Balance improvements in older women: effects of exercise training [J]. Phys Ther, 1993, 73(4): 254-62; discussion 63-5. |
| 34 | KIENLE G S, WERTHMANN P G, GROTEJOHANN B, et al. A multi-centre, parallel-group, randomised controlled trial to assess the efficacy and safety of eurythmy therapy and tai chi in comparison with standard care in chronically ill elderly patients with increased risk of falling (ENTAiER): a trial protocol [J]. BMC Geriatr, 2020, 20(1): 108. |
| 35 | KIM C Y, JE H D, JEONG H, et al. Effects of Tai Chi versus Taekkyon on balance, lower-extremity strength, and gait ability in community-dwelling older women: A single-blinded randomized clinical trial [J]. J Back Musculoskelet Rehabil, 2020, 33(1): 41-8. |
| 36 | KOHN J N, LOBO J D, TROYER E A, et al. Tai Chi versus health education as a frailty intervention for community-dwelling older adults with hypertension [J]. Aging Clin Exp Res, 2023, 35(10): 2051-60. |
| 37 | KOHN J N, LOBO J D, TROYER E A, et al. Tai chi or health education for older adults with hypertension: effects on mental health and psychological resilience to COVID-19 [J]. Aging Ment Health, 2023, 27(3): 496-504. |
| 38 | KUTNER N G, BARNHART H, WOLF S L, et al. Self-report benefits of Tai Chi practice by older adults [J]. J Gerontol B Psychol Sci Soc Sci, 1997, 52(5): P242-6. |
| 39 | LAM L C, CHAU R C, WONG B M, et al. Interim follow-up of a randomized controlled trial comparing Chinese style mind body (Tai Chi) and stretching exercises on cognitive function in subjects at risk of progressive cognitive decline [J]. Int J Geriatr Psychiatry, 2011, 26(7): 733-40. |
| 40 | LAM L C, CHAU R C, WONG B M, et al. A 1-year randomized controlled trial comparing mind body exercise (Tai Chi) with stretching and toning exercise on cognitive function in older Chinese adults at risk of cognitive decline [J]. J Am Med Dir Assoc, 2012, 13(6): 568.e15-20. |
| 41 | LARKEY L K, JAMES T, HAN S, JAMES D L. Pilot study of Qigong/Tai Chi Easy acute effects of meditative movement, breath focus and "flow" on blood pressure, mood and oxytocin in older adults [J]. Complement Ther Med, 2023, 72: 102918. |
| 42 | LAVRETSKY H, ALSTEIN L L, OLMSTEAD R E, et al. Complementary use of tai chi chih augments escitalopram treatment of geriatric depression: a randomized controlled trial [J]. Am J Geriatr Psychiatry, 2011, 19(10): 839-50. |
| 43 | LAVRETSKY H, MILILLO M M, KILPATRICK L, et al. A Randomized Controlled Trial of Tai Chi Chih or Health Education for Geriatric Depression [J]. Am J Geriatr Psychiatry, 2022, 30(3): 392-403. |
| 44 | LEE K Y, HUI-CHAN C W, TSANG W W. The effects of practicing sitting Tai Chi on balance control and eye-hand coordination in the older adults: a randomized controlled trial [J]. Disabil Rehabil, 2015, 37(9): 790-4. |
| 45 | LEE L Y, LEE D T, WOO J. Effect of Tai Chi on state self-esteem and health-related quality of life in older Chinese residential care home residents [J]. J Clin Nurs, 2007, 16(8): 1580-2. |
| 46 | LEE T L, SHERMAN K J, HAWKES R J, et al. The Benefits of T'ai Chi for Older Adults with Chronic Back Pain: A Qualitative Study [J]. J Altern Complement Med, 2020, 26(6): 456-62. |
| 47 | LELARD T, DOUTRELLOT P L, DAVID P, AHMAIDI S. Effects of a 12-week Tai Chi Chuan program versus a balance training program on postural control and walking ability in older people [J]. Arch Phys Med Rehabil, 2010, 91(1): 9-14. |
| 48 | LEUNG L Y, CHAN A W, SIT J W, et al. Tai Chi in Chinese adults with metabolic syndrome: A pilot randomized controlled trial [J]. Complement Ther Med, 2019, 46: 54-61. |
| 49 | LI F, FISHER K J, HARMER P, et al. Tai chi and self-rated quality of sleep and daytime sleepiness in older adults: a randomized controlled trial [J]. J Am Geriatr Soc, 2004, 52(6): 892-900. |
| 50 | LI F, HARMER P, CHOU L S. Dual-Task Walking Capacity Mediates Tai Ji Quan Impact on Physical and Cognitive Function [J]. Med Sci Sports Exerc, 2019, 51(11): 2318-24. |
| 51 | LI F, HARMER P, ECKSTROM E, et al. Effectiveness of Tai Ji Quan vs Multimodal and Stretching Exercise Interventions for Reducing Injurious Falls in Older Adults at High Risk of Falling: Follow-up Analysis of a Randomized Clinical Trial [J]. JAMA Netw Open, 2019, 2(2): e188280. |
| 52 | LI F, HARMER P, ECKSTROM E, et al. Clinical Effectiveness of Cognitively Enhanced Tai Ji Quan Training on Global Cognition and Dual-Task Performance During Walking in Older Adults With Mild Cognitive Impairment or Self-Reported Memory Concerns : A Randomized Controlled Trial [J]. Ann Intern Med, 2023, 176(11): 1498-507. |
| 53 | LI F, HARMER P, FISHER K J, MCAULEY E. Tai Chi: improving functional balance and predicting subsequent falls in older persons [J]. Med Sci Sports Exerc, 2004, 36(12): 2046-52. |
| 54 | LI F, HARMER P, FISHER K J, et al. Tai Chi and fall reductions in older adults: a randomized controlled trial [J]. J Gerontol A Biol Sci Med Sci, 2005, 60(2): 187-94. |
| 55 | LI F, HARMER P, FITZGERALD K, et al. Effectiveness of a Therapeutic Tai Ji Quan Intervention vs a Multimodal Exercise Intervention to Prevent Falls Among Older Adults at High Risk of Falling: A Randomized Clinical Trial [J]. JAMA Intern Med, 2018, 178(10): 1301-10. |
| 56 | LI F, HARMER P, FITZGERALD K, WINTERS-STONE K. A cognitively enhanced online Tai Ji Quan training intervention for community-dwelling older adults with mild cognitive impairment: A feasibility trial [J]. BMC Geriatr, 2022, 22(1): 76. |
| 57 | LI F, HARMER P, MCAULEY E, et al. An evaluation of the effects of Tai Chi exercise on physical function among older persons: a randomized contolled trial [J]. Ann Behav Med, 2001, 23(2): 139-46. |
| 58 | LI F, HARMER P, MCAULEY E, et al. Tai Chi, self-efficacy, and physical function in the elderly [J]. Prev Sci, 2001, 2(4): 229-39. |
| 59 | LI F, HARMER P, VOIT J, CHOU L S. Implementing an Online Virtual Falls Prevention Intervention During a Public Health Pandemic for Older Adults with Mild Cognitive Impairment: A Feasibility Trial [J]. Clin Interv Aging, 2021, 16: 973-83. |
| 60 | LI J X, XU D Q, HONG Y. Effects of 16-week Tai Chi intervention on postural stability and proprioception of knee and ankle in older people [J]. Age Ageing, 2008, 37(5): 575-8. |
| 61 | LI X, SI H, CHEN Y, et al. Effects of fitness qigong and tai chi on middle-aged and elderly patients with type 2 diabetes mellitus [J]. PLoS One, 2020, 15(12): e0243989. |
| 62 | LIANG I J, PERKIN O J, MCGUIGAN P M, et al. Feasibility and Acceptability of Home-Based Exercise Snacking and Tai Chi Snacking Delivered Remotely to Self-Isolating Older Adults During COVID-19 [J]. J Aging Phys Act, 2022, 30(1): 33-43. |
| 63 | LIAO S J, CHONG M C, TAN M P, CHUA Y P. Tai Chi with music improves quality of life among community-dwelling older persons with mild to moderate depressive symptoms: A cluster randomized controlled trial [J]. Geriatr Nurs, 2019, 40(2): 154-9. |
| 64 | LIAO S J, TAN M P, CHONG M C, CHUA Y P. The Impact of Combined Music and Tai Chi on Depressive Symptoms Among Community-Dwelling Older Persons: A Cluster Randomized Controlled Trial [J]. Issues Ment Health Nurs, 2018, 39(5): 398-402. |
| 65 | LIN M, LIU W, MA C, et al. Tai Chi-Induced Exosomal LRP1 is Associated With Memory Function and Hippocampus Plasticity in aMCI Patients [J]. Am J Geriatr Psychiatry, 2024, 32(10): 1215-30. |
| 66 | LIN S F, SUNG H C, LI T L, et al. The effects of Tai-Chi in conjunction with thera-band resistance exercise on functional fitness and muscle strength among community-based older people [J]. J Clin Nurs, 2015, 24(9-10): 1357-66. |
| 67 | LIPSITZ L A, MACKLIN E A, TRAVISON T G, et al. A Cluster Randomized Trial of Tai Chi vs Health Education in Subsidized Housing: The MI-WiSH Study [J]. J Am Geriatr Soc, 2019, 67(9): 1812-9. |
| 68 | LIU J, XIE H, LIU M, et al. The Effects of Tai Chi on Heart Rate Variability in Older Chinese Individuals with Depression [J]. Int J Environ Res Public Health, 2018, 15(12). |
| 69 | LIU X, HUANG G, CHEN P, et al. Comparative effects of Yi Jin Jing versus Tai Chi exercise training on benign prostatic hyperplasia-related outcomes in older adults: study protocol for a randomized controlled trial [J]. Trials, 2016, 17(1): 319. |
| 70 | LU X, HUI-CHAN C W, TSANG W W. Effects of Tai Chi training on arterial compliance and muscle strength in female seniors: a randomized clinical trial [J]. Eur J Prev Cardiol, 2013, 20(2): 238-45. |
| 71 | LU X, SIU K C, FU S N, et al. Tai Chi practitioners have better postural control and selective attention in stepping down with and without a concurrent auditory response task [J]. Eur J Appl Physiol, 2013, 113(8): 1939-45. |
| 72 | LU X, SIU K C, FU S N, et al. Effects of Tai Chi training on postural control and cognitive performance while dual tasking - a randomized clinical trial [J]. J Complement Integr Med, 2016, 13(2): 181-7. |
| 73 | MA C, ZHOU W, TANG Q, HUANG S. The impact of group-based Tai chi on health-status outcomes among community-dwelling older adults with hypertension [J]. Heart Lung, 2018, 47(4): 337-44. |
| 74 | MA Y, GOW B J, SONG R, et al. Long-term Tai Chi practice in older adults is associated with "younger" functional abilities [J]. Aging Cell, 2024, 23(1): e14023. |
| 75 | MA Y, WU C W, PENG C K, et al. Complexity-Based Measures of Heart Rate Dynamics in Older Adults Following Long- and Short-Term Tai Chi Training: Cross-sectional and Randomized Trial Studies [J]. Sci Rep, 2019, 9(1): 7500. |
| 76 | MANOR B, LOUGH M, GAGNON M M, et al. Functional benefits of tai chi training in senior housing facilities [J]. J Am Geriatr Soc, 2014, 62(8): 1484-9. |
| 77 | MAO M, MERCER V S, LI F, et al. The effect of Tai Chi lower extremity exercise on the balance control of older adults in assistant living communities [J]. BMC Complement Med Ther, 2024, 24(1): 112. |
| 78 | MASTEL-SMITH B, DUKE G, HE Z. A Pilot Randomized Controlled Trial Examining the Effects of Tai Chi and Electronic Tablet Use on Older Adults' Cognition and Health [J]. J Holist Nurs, 2019, 37(2): 163-74. |
| 79 | MCGIBBON C A, KREBS D E, PARKER S W, et al. Tai Chi and vestibular rehabilitation improve vestibulopathic gait via different neuromuscular mechanisms: preliminary report [J]. BMC Neurol, 2005, 5(1): 3. |
| 80 | MEROM D, CUMMING R, MATHIEU E, et al. Can social dancing prevent falls in older adults? a protocol of the Dance, Aging, Cognition, Economics (DAnCE) fall prevention randomised controlled trial [J]. BMC Public Health, 2013, 13: 477. |
| 81 | MOAWD S A, NAMBI G, ALRAWAILI S M, et al. Analyzing the influence of the combination of monochromatic infrared energy and tai chi exercise improve balance in community-dwelling older adults with lower-extremity disease: a double-blinded randomized controlled study [J]. Eur Rev Med Pharmacol Sci, 2022, 26(21): 7788-96. |
| 82 | MOORE A A, LAKE J E, GLASNER S, et al. Establishing the feasibility, acceptability and preliminary efficacy of a multi-component behavioral intervention to reduce pain and substance use and improve physical performance in older persons living with HIV [J]. J Subst Abuse Treat, 2019, 100: 29-38. |
| 83 | MORAWIN B, TYLUTKA A, CHMIELOWIEC J, ZEMBRON-LACNY A. Circulating Mediators of Apoptosis and Inflammation in Aging; Physical Exercise Intervention [J]. Int J Environ Res Public Health, 2021, 18(6). |
| 84 | NI G X, SONG L, YU B, et al. Tai chi improves physical function in older Chinese women with knee osteoarthritis [J]. J Clin Rheumatol, 2010, 16(2): 64-7. |
| 85 | NI M, MOONEY K, RICHARDS L, et al. Comparative impacts of Tai Chi, balance training, and a specially-designed yoga program on balance in older fallers [J]. Arch Phys Med Rehabil, 2014, 95(9): 1620-8.e30. |
| 86 | NICHOLSON V P, MCKEAN M R, BURKETT B J. Twelve weeks of BodyBalance® training improved balance and functional task performance in middle-aged and older adults [J]. Clin Interv Aging, 2014, 9: 1895-904. |
| 87 | NISSIM M, HUTZLER Y, GOLDSTEIN A. A walk on water: comparing the influence of Ai Chi and Tai Chi on fall risk and verbal working memory in ageing people with intellectual disabilities - a randomised controlled trial [J]. J Intellect Disabil Res, 2019, 63(6): 603-13. |
| 88 | NORADECHANUNT C, WORSLEY A, GROELLER H. Thai Yoga improves physical function and well-being in older adults: A randomised controlled trial [J]. J Sci Med Sport, 2017, 20(5): 494-501. |
| 89 | NOWALK M P, PRENDERGAST J M, BAYLES C M, et al. A randomized trial of exercise programs among older individuals living in two long-term care facilities: the FallsFREE program [J]. J Am Geriatr Soc, 2001, 49(7): 859-65. |
| 90 | NYMAN S R, CASEY C, POLMAN R. Psychometric Properties of the ICECAP-O Quality of Life Measurement Tool When Self-reported by Community-dwelling Older People With Mild and Moderate Dementia [J]. Alzheimer Dis Assoc Disord, 2021, 35(4): 356-9. |
| 91 | NYMAN S R, HAYWARD C, INGRAM W, et al. A randomised controlled trial comparing the effectiveness of tai chi alongside usual care with usual care alone on the postural balance of community-dwelling people with dementia: protocol for the TACIT trial (TAi ChI for people with demenTia) [J]. BMC Geriatr, 2018, 18(1): 263. |
| 92 | PALUMBO M V, WU G, SHANER-MCRAE H, et al. Tai Chi for older nurses: a workplace wellness pilot study [J]. Appl Nurs Res, 2012, 25(1): 54-9. |
| 93 | PAN C, WANG X, DENG Y, et al. Efficacy of mindfulness-based intervention ('mindfulness-based joyful sleep') in young and middle-aged individuals with insomnia using a biomarker of inflammatory responses: a prospective protocol of a randomised controlled trial in China [J]. BMJ Open, 2019, 9(7): e027061. |
| 94 | PAYNE K B, BRAZIL C K, APEL M, BAILEY H. Knowledge-based intervention improves older adult recognition memory for novel activity, but not event segmentation or temporal order memory [J]. Sci Rep, 2023, 13(1): 18679. |
| 95 | PLISKE G, EMMERMACHER P, WEINBEER V, WITTE K. Changes in dual-task performance after 5 months of karate and fitness training for older adults to enhance fall prevention [J]. Aging Clin Exp Res, 2016, 28(6): 1179-86. |
| 96 | PLUCHINO A, LEE S Y, ASFOUR S, et al. Pilot study comparing changes in postural control after training using a video game balance board program and 2 standard activity-based balance intervention programs [J]. Arch Phys Med Rehabil, 2012, 93(7): 1138-46. |
| 97 | QI M, MOYLE W, JONES C, WEEKS B. Feasibility of a Tai Chi with Thera-Band Training Program: A Pilot Study [J]. Int J Environ Res Public Health, 2020, 17(22). |
| 98 | QUIGLEY P A, BULAT T, SCHULZ B, et al. Exercise interventions, gait, and balance in older subjects with distal symmetric polyneuropathy: a three-group randomized clinical trial [J]. Am J Phys Med Rehabil, 2014, 93(1): 1-12; quiz 3-6. |
| 99 | RAMESHKUMAR R, LARKEY L, ALPERIN K, et al. Study design exploring Qigong and Tai Chi Easy (QTC) on cardiometabolic risk factors [J]. Contemp Clin Trials, 2022, 118: 106793. |
| 100 | REDWINE L S, PUNG M A, WILSON K, et al. An exploratory randomized sub-study of light-to-moderate intensity exercise on cognitive function, depression symptoms and inflammation in older adults with heart failure [J]. J Psychosom Res, 2020, 128: 109883. |
| 101 | RIKKONEN T, SUND R, KOIVUMAA-HONKANEN H, et al. Effectiveness of exercise on fall prevention in community-dwelling older adults: a 2-year randomized controlled study of 914 women [J]. Age Ageing, 2023, 52(4). |
| 102 | ROA W, BRASHER P M, BAUMAN G, et al. Abbreviated course of radiation therapy in older patients with glioblastoma multiforme: a prospective randomized clinical trial [J]. J Clin Oncol, 2004, 22(9): 1583-8. |
| 103 | ROSADO-PéREZ J, SANTIAGO-OSORIO E, ORTIZ R, MENDOZA-NúñEZ V M. Tai chi diminishes oxidative stress in Mexican older adults [J]. J Nutr Health Aging, 2012, 16(7): 642-6. |
| 104 | SADEGHIAN F, ZOLAKTAF V, SHIGEMATSU R. A comparison between effects of Square-Stepping Exercise and Tai Chi Chuan on functional fitness and fear of falling in older women [J]. Aging Clin Exp Res, 2023, 35(4): 827-34. |
| 105 | SARAVANAKUMAR P, HIGGINS I J, VAN DER RIET P J, et al. The influence of tai chi and yoga on balance and falls in a residential care setting: A randomised controlled trial [J]. Contemp Nurse, 2014, 48(1): 76-87. |
| 106 | SATTIN R W, EASLEY K A, WOLF S L, et al. Reduction in fear of falling through intense tai chi exercise training in older, transitionally frail adults [J]. J Am Geriatr Soc, 2005, 53(7): 1168-78. |
| 107 | SHEN C L, CHYU M C, YEH J K, et al. Green tea polyphenols and Tai Chi for bone health: designing a placebo-controlled randomized trial [J]. BMC Musculoskelet Disord, 2009, 10: 110. |
| 108 | SHERMAN K J, WELLMAN R D, HAWKES R J, et al. T'ai Chi for Chronic Low Back Pain in Older Adults: A Feasibility Trial [J]. J Altern Complement Med, 2020, 26(3): 176-89. |
| 109 | SIDDARTH P, ABIKENARI M, GRZENDA A, et al. Inflammatory Markers of Geriatric Depression Response to Tai Chi or Health Education Adjunct Interventions [J]. Am J Geriatr Psychiatry, 2023, 31(1): 22-32. |
| 110 | SIU P M, YU A P, CHIN E C, et al. Effects of Tai Chi or Conventional Exercise on Central Obesity in Middle-Aged and Older Adults : A Three-Group Randomized Controlled Trial [J]. Ann Intern Med, 2021, 174(8): 1050-7. |
| 111 | SIU P M, YU A P, TAM B T, et al. Effects of Tai Chi or Exercise on Sleep in Older Adults With Insomnia: A Randomized Clinical Trial [J]. JAMA Netw Open, 2021, 4(2): e2037199. |
| 112 | SMITH-RAY R L, MAKOWSKI-WOIDAN B, HUGHES S L. A randomized trial to measure the impact of a community-based cognitive training intervention on balance and gait in cognitively intact Black older adults [J]. Health Educ Behav, 2014, 41(1 Suppl): 62s-9s. |
| 113 | SOLIANIK R, BRAZAITIS M, ČEKANAUSKAITĖ-KRUŠNAUSKIENĖ A. Tai chi effects on balance in older adults: the role of sustained attention and myokines [J]. J Sports Med Phys Fitness, 2022, 62(11): 1512-8. |
| 114 | SOLIANIK R, MICKEVIČIENĖ D, ŽLIBINAITĖ L, ČEKANAUSKAITĖ A. Tai chi improves psychoemotional state, cognition, and motor learning in older adults during the COVID-19 pandemic [J]. Exp Gerontol, 2021, 150: 111363. |
| 115 | SON N K, RYU Y U, JEONG H W, et al. Comparison of 2 Different Exercise Approaches: Tai Chi Versus Otago, in Community-Dwelling Older Women [J]. J Geriatr Phys Ther, 2016, 39(2): 51-7. |
| 116 | SONG R, LEE E O, LAM P, BAE S C. Effects of tai chi exercise on pain, balance, muscle strength, and perceived difficulties in physical functioning in older women with osteoarthritis: a randomized clinical trial [J]. J Rheumatol, 2003, 30(9): 2039-44. |
| 117 | SONG R, LEE E O, LAM P, BAE S C. Effects of a Sun-style Tai Chi exercise on arthritic symptoms, motivation and the performance of health behaviors in women with osteoarthritis [J]. Taehan Kanho Hakhoe Chi, 2007, 37(2): 249-56. |
| 118 | SONG R, ROBERTS B L, LEE E O, et al. A randomized study of the effects of t'ai chi on muscle strength, bone mineral density, and fear of falling in women with osteoarthritis [J]. J Altern Complement Med, 2010, 16(3): 227-33. |
| 119 | SU Z, ZHAO J, WANG N, et al. Effects of Weighted Tai Chi on Leg Strength of Older Adults [J]. J Am Geriatr Soc, 2015, 63(10): 2208-10. |
| 120 | SUKSOM D, SIRIPATT A, LAPO P, PATUMRAJ S. Effects of two modes of exercise on physical fitness and endothelial function in the elderly: exercise with a flexible stick versus Tai Chi [J]. J Med Assoc Thai, 2011, 94(1): 123-32. |
| 121 | SUN J, BUYS N. Community-Based Mind-Body Meditative Tai Chi Program and Its Effects on Improvement of Blood Pressure, Weight, Renal Function, Serum Lipoprotein, and Quality of Life in Chinese Adults With Hypertension [J]. Am J Cardiol, 2015, 116(7): 1076-81. |
| 122 | SUNGKARAT S, BORIPUNTAKUL S, CHATTIPAKORN N, et al. Effects of Tai Chi on Cognition and Fall Risk in Older Adults with Mild Cognitive Impairment: A Randomized Controlled Trial [J]. J Am Geriatr Soc, 2017, 65(4): 721-7. |
| 123 | SUNGKARAT S, BORIPUNTAKUL S, KUMFU S, et al. Tai Chi Improves Cognition and Plasma BDNF in Older Adults With Mild Cognitive Impairment: A Randomized Controlled Trial [J]. Neurorehabil Neural Repair, 2018, 32(2): 142-9. |
| 124 | TAJIK A, REJEH N, HERAVI-KARIMOOI M, et al. The effect of Tai Chi on quality of life in male older people: A randomized controlled clinical trial [J]. Complement Ther Clin Pract, 2018, 33: 191-6. |
| 125 | TAO J, RAO T, LIN L, et al. Evaluation of Tai Chi Yunshou exercises on community-based stroke patients with balance dysfunction: a study protocol of a cluster randomized controlled trial [J]. BMC Complement Altern Med, 2015, 15: 31. |
| 126 | TAYLOR D, HALE L, SCHLUTER P, et al. Effectiveness of tai chi as a community-based falls prevention intervention: a randomized controlled trial [J]. J Am Geriatr Soc, 2012, 60(5): 841-8. |
| 127 | TAYLOR-PILIAE R E, BOROS D, COULL B M. Strategies to improve recruitment and retention of older stroke survivors to a randomized clinical exercise trial [J]. J Stroke Cerebrovasc Dis, 2014, 23(3): 462-8. |
| 128 | TAYLOR-PILIAE R E, HOKE T M, HEPWORTH J T, et al. Effect of Tai Chi on physical function, fall rates and quality of life among older stroke survivors [J]. Arch Phys Med Rehabil, 2014, 95(5): 816-24. |
| 129 | TAYLOR-PILIAE R E, NEWELL K A, CHERIN R, et al. Effects of Tai Chi and Western exercise on physical and cognitive functioning in healthy community-dwelling older adults [J]. J Aging Phys Act, 2010, 18(3): 261-79. |
| 130 | TIEDEMANN A, O'ROURKE S, SESTO R, SHERRINGTON C. A 12-week Iyengar yoga program improved balance and mobility in older community-dwelling people: a pilot randomized controlled trial [J]. J Gerontol A Biol Sci Med Sci, 2013, 68(9): 1068-75. |
| 131 | TOUSIGNANT M, CORRIVEAU H, KAIRY D, et al. Tai Chi-based exercise program provided via telerehabilitation compared to home visits in a post-stroke population who have returned home without intensive rehabilitation: study protocol for a randomized, non-inferiority clinical trial [J]. Trials, 2014, 15: 42. |
| 132 | TOUSIGNANT M, CORRIVEAU H, ROY P M, et al. Efficacy of supervised Tai Chi exercises versus conventional physical therapy exercises in fall prevention for frail older adults: a randomized controlled trial [J]. Disabil Rehabil, 2013, 35(17): 1429-35. |
| 133 | TOUSIGNANT M, CORRIVEAU H, ROY P M, et al. The effect of supervised Tai Chi intervention compared to a physiotherapy program on fall-related clinical outcomes: a randomized clinical trial [J]. Disabil Rehabil, 2012, 34(3): 196-201. |
| 134 | TSANG T, ORR R, LAM P, et al. Effects of Tai Chi on glucose homeostasis and insulin sensitivity in older adults with type 2 diabetes: a randomised double-blind sham-exercise-controlled trial [J]. Age Ageing, 2008, 37(1): 64-71. |
| 135 | TSANG T, ORR R, LAM P, et al. Health benefits of Tai Chi for older patients with type 2 diabetes: the "Move It For Diabetes study"--a randomized controlled trial [J]. Clin Interv Aging, 2007, 2(3): 429-39. |
| 136 | TSE M M, LEE P H, NG S M, et al. Peer volunteers in an integrative pain management program for frail older adults with chronic pain: study protocol for a randomized controlled trial [J]. Trials, 2014, 15: 205. |
| 137 | VALLABHAJOSULA S, ROBERTS B L, HASS C J. Tai chi intervention improves dynamic postural control during gait initiation in older adults: a pilot study [J]. J Appl Biomech, 2014, 30(6): 697-706. |
| 138 | VOUKELATOS A, CUMMING R G, LORD S R, RISSEL C. A randomized, controlled trial of tai chi for the prevention of falls: the Central Sydney tai chi trial [J]. J Am Geriatr Soc, 2007, 55(8): 1185-91. |
| 139 | WANG X, HAN Y, LI H, et al. Impact of a Precision Intervention for Vascular Health in Middle-Aged and Older Postmenopausal Women Using Polar Heart Rate Sensors: A 24-Week RCT Study Based on the New Compilation of Tai Chi (Bafa Wubu) [J]. Sensors (Basel), 2024, 24(17). |
| 140 | WANG X, HOU M, CHEN S, et al. Effects of tai chi on postural control during dual-task stair negotiation in knee osteoarthritis: a randomised controlled trial protocol [J]. BMJ Open, 2020, 10(1): e033230. |
| 141 | WANG Y, LUO B, WU X, et al. Comparison of the effects of Tai Chi and general aerobic exercise on weight, blood pressure and glycemic control among older persons with depressive symptoms: a randomized trial [J]. BMC Geriatr, 2022, 22(1): 401. |
| 142 | WATERS D L, HALE L, GRANT A M, et al. Osteoporosis and gait and balance disturbances in older sarcopenic obese New Zealanders [J]. Osteoporos Int, 2010, 21(2): 351-7. |
| 143 | WAYNE P M, GAGNON M M, MACKLIN E A, et al. The Mind Body-Wellness in Supportive Housing (Mi-WiSH) study: Design and rationale of a cluster randomized controlled trial of Tai Chi in senior housing [J]. Contemp Clin Trials, 2017, 60: 96-104. |
| 144 | WAYNE P M, GOW B J, COSTA M D, et al. Complexity-Based Measures Inform Effects of Tai Chi Training on Standing Postural Control: Cross-Sectional and Randomized Trial Studies [J]. PLoS One, 2014, 9(12): e114731. |
| 145 | WAYNE P M, GOW B J, HOU F, et al. Tai Chi training's effect on lower extremity muscle co-contraction during single- and dual-task gait: Cross-sectional and randomized trial studies [J]. PLoS One, 2021, 16(1): e0242963. |
| 146 | WINTERS-STONE K M, HORAK F, DIECKMANN N F, et al. GET FIT: A Randomized Clinical Trial of Tai Ji Quan Versus Strength Training for Fall Prevention After Chemotherapy in Older, Postmenopausal Women Cancer Survivors [J]. J Clin Oncol, 2023, 41(18): 3384-96. |
| 147 | WINTERS-STONE K M, LI F, HORAK F, et al. Comparison of tai chi vs. strength training for fall prevention among female cancer survivors: study protocol for the GET FIT trial [J]. BMC Cancer, 2012, 12: 577. |
| 148 | WINTERS-STONE K M, STOYLES S A, DIECKMANN N F, et al. Can strength training or tai ji quan training reduce frailty in postmenopausal women treated with chemotherapy? A secondary data analysis of the GET FIT trial [J]. J Cancer Surviv, 2024, 18(4): 1179-89. |
| 149 | WOLF S L, BARNHART H X, ELLISON G L, COOGLER C E. The effect of Tai Chi Quan and computerized balance training on postural stability in older subjects. Atlanta FICSIT Group. Frailty and Injuries: Cooperative Studies on Intervention Techniques [J]. Phys Ther, 1997, 77(4): 371-81; discussion 82-4. |
| 150 | WOLF S L, BARNHART H X, KUTNER N G, et al. Reducing frailty and falls in older persons: an investigation of Tai Chi and computerized balance training. Atlanta FICSIT Group. Frailty and Injuries: Cooperative Studies of Intervention Techniques [J]. J Am Geriatr Soc, 1996, 44(5): 489-97. |
| 151 | WOLF S L, BARNHART H X, KUTNER N G, et al. Selected as the best paper in the 1990s: Reducing frailty and falls in older persons: an investigation of tai chi and computerized balance training [J]. J Am Geriatr Soc, 2003, 51(12): 1794-803. |
| 152 | WOLF S L, O'GRADY M, EASLEY K A, et al. The influence of intense Tai Chi training on physical performance and hemodynamic outcomes in transitionally frail, older adults [J]. J Gerontol A Biol Sci Med Sci, 2006, 61(2): 184-9. |
| 153 | WOLF S L, SATTIN R W, KUTNER M, et al. Intense tai chi exercise training and fall occurrences in older, transitionally frail adults: a randomized, controlled trial [J]. J Am Geriatr Soc, 2003, 51(12): 1693-701. |
| 154 | WOLF S L, SATTIN R W, O'GRADY M, et al. A study design to investigate the effect of intense Tai Chi in reducing falls among older adults transitioning to frailty [J]. Control Clin Trials, 2001, 22(6): 689-704. |
| 155 | WOLFSON L, WHIPPLE R, DERBY C, et al. Balance and strength training in older adults: intervention gains and Tai Chi maintenance [J]. J Am Geriatr Soc, 1996, 44(5): 498-506. |
| 156 | WOO S C, CHEN M Y, CHEN L K, LIU C Y. Effectiveness of Resistance Band Use in Conjunction With Tai Chi Among Older Adults With Prefrailty to Improve Functional Fitness, Quality of Life, and Heart Rate Variability [J]. J Gerontol Nurs, 2024, 50(5): 19-26. |
| 157 | WU Y, SENK C, COLL P, et al. A comparison of two Tai Chi interventions tailored for different health outcomes [J]. Complement Ther Med, 2021, 59: 102731. |
| 158 | XIAO C M. Effects of long-term tai chi ball practice on balance performance in older adults [J]. J Am Geriatr Soc, 2014, 62(5): 984-5. |
| 159 | XIAO C M, ZHUANG Y C. Effects of Tai Chi ball on balance and physical function in older adults with type 2 diabetes mellitus [J]. J Am Geriatr Soc, 2015, 63(1): 176-7. |
| 160 | XU F, LETENDRE J, BEKKE J, et al. Impact of a program of Tai Chi plus behaviorally based dietary weight loss on physical functioning and coronary heart disease risk factors: a community-based study in obese older women [J]. J Nutr Gerontol Geriatr, 2015, 34(1): 50-65. |
| 161 | XU Y, ZHU J, LIU H, et al. Effects of Tai Chi combined with tDCS on cognitive function in patients with MCI: a randomized controlled trial [J]. Front Public Health, 2023, 11: 1199246. |
| 162 | YAN Z W, YANG Z, YANG J H, et al. Comparison between Tai Chi and square dance on the antihypertensive effect and cardiovascular disease risk factors in patients with essential hypertension: a 12-week randomized controlled trial [J]. J Sports Med Phys Fitness, 2022, 62(11): 1568-75. |
| 163 | YANG Y, HAO Y L, TIAN W J, et al. The effectiveness of Tai Chi for patients with Parkinson's disease: study protocol for a randomized controlled trial [J]. Trials, 2015, 16: 111. |
| 164 | YANG Y, VERKUILEN J V, ROSENGREN K S, et al. Effect of combined Taiji and Qigong training on balance mechanisms: a randomized controlled trial of older adults [J]. Med Sci Monit, 2007, 13(8): Cr339-48. |
| 165 | YıLDıRıM P, OFLUOGLU D, AYDOGAN S, AKYUZ G. Tai Chi vs. combined exercise prescription: A comparison of their effects on factors related to falls [J]. J Back Musculoskelet Rehabil, 2016, 29(3): 493-501. |
| 166 | YOU T, OGAWA E F, THAPA S, et al. Effects of Tai Chi on beta endorphin and inflammatory markers in older adults with chronic pain: an exploratory study [J]. Aging Clin Exp Res, 2020, 32(7): 1389-92. |
| 167 | YOU T, OGAWA E F, THAPA S, et al. Tai Chi for older adults with chronic multisite pain: a randomized controlled pilot study [J]. Aging Clin Exp Res, 2018, 30(11): 1335-43. |
| 168 | YOUNG D K. Multicomponent intervention combining a cognitive stimulation group and tai chi to reduce cognitive decline among community-dwelling older adults with probable dementia: A multi-center, randomized controlled trial [J]. Dementia (London), 2020, 19(6): 2073-89. |
| 169 | YOUNG D R, APPEL L J, JEE S, MILLER E R, 3RD. The effects of aerobic exercise and T'ai Chi on blood pressure in older people: results of a randomized trial [J]. J Am Geriatr Soc, 1999, 47(3): 277-84. |
| 170 | YOUNG K D, KWOK C T, NG Y P, et al. Multicomponent Intervention on Improving the Cognitive Ability of Older Adults with Mild Cognitive Impairment: A Pilot Randomized Controlled Trial [J]. J Gerontol Soc Work, 2024, 67(4): 492-514. |
| 171 | YU A P, CHIN E C, YU D J, et al. Tai Chi versus conventional exercise for improving cognitive function in older adults: a pilot randomized controlled trial [J]. Sci Rep, 2022, 12(1): 8868. |
| 172 | YU J, KIM J. [Effects of a physical activity program using exergame with elderly women] [J]. J Korean Acad Nurs, 2015, 45(1): 84-96. |
| 173 | ZHANG J G, ISHIKAWA-TAKATA K, YAMAZAKI H, et al. The effects of Tai Chi Chuan on physiological function and fear of falling in the less robust elderly: an intervention study for preventing falls [J]. Arch Gerontol Geriatr, 2006, 42(2): 107-16. |
| 174 | ZHAO Y, CHUNG P K, TONG T K. Effectiveness of a Community-Based Exercise Program on Balance Performance and Fear of Falling in Older Nonfallers at Risk for Falling: A Randomized, Controlled Study [J]. J Aging Phys Act, 2016, 24(4): 516-24. |
| 175 | ZHAO Y, CHUNG P K, TONG T K. Effectiveness of a balance-focused exercise program for enhancing functional fitness of older adults at risk of falling: A randomised controlled trial [J]. Geriatr Nurs, 2017, 38(6): 491-7. |
| 176 | ZHENG G, XIONG Z, ZHENG X, et al. Subjective perceived impact of Tai Chi training on physical and mental health among community older adults at risk for ischemic stroke: a qualitative study [J]. BMC Complement Altern Med, 2017, 17(1): 221. |
| 177 | ZHENG G, ZHENG X, LI J, et al. Effects of Tai Chi on Cerebral Hemodynamics and Health-Related Outcomes in Older Community Adults at Risk of Ischemic Stroke: A Randomized Controlled Trial [J]. J Aging Phys Act, 2019, 27(5): 678–87. |
| 178 | ZHENG G H, ZHENG X, LI J Z, et al. Effect of Tai Chi on Cardiac and Static Pulmonary Function in Older Community-Dwelling Adults at Risk of Ischemic Stroke: A Randomized Controlled Trial [J]. Chin J Integr Med, 2019, 25(8): 582-9. |
| 179 | ZHU R, WANG W, ZHAO L, MAO S. Comparisons of tai chi and Iyengar yoga intervention effects on muscle strength, balance, and confidence in balance [J]. J Sports Med Phys Fitness, 2021, 61(10): 1333-8. |
| 180 | ZHUANG J, HUANG L, WU Y, ZHANG Y. The effectiveness of a combined exercise intervention on physical fitness factors related to falls in community-dwelling older adults [J]. Clin Interv Aging, 2014, 9: 131-40. |
